# Quillaic acid and echinocystic acid attenuate atherosclerosis: evidence from *in vitro* and *in vivo* studies

**DOI:** 10.7717/peerj.21907

**Published:** 2026-11-12

**Authors:** Jian Ai, Xin Tang, Yongkang Zhou, Fei Liu, Bingyong Mao, Qiuxiang Zhang, Jianxin Zhao, Wei Chen, Shumao Cui

**Affiliations:** 1 State Key Laboratory of Food Science and Resources, Jiangnan University, Wuxi, China; 2 School of Food Science and Technology, Jiangnan University, Wuxi, China; 3 National Engineering Research Center for Functional Food, Jiangnan University, Wuxi, China; 4 Wuxi Institute for Specialized Nutrition and Health Co., Ltd., Wuxi, China

**Keywords:** Natural products, Quillaic acid, Echinocystic acid, Atherosclerosis

## Abstract

**Background:**

Atherosclerosis (AS) is an inflammatory disease characterized by the formation of lipid-laden lesions in arterial walls. Quillaic acid (QA) and echinocystic acid (EA) are natural pentacyclic triterpenes with diverse biological activities. However, whether QA and EA can ameliorate AS has not been reported.

**Methods:**

In this study, we first investigated the effects of QA and EA in reducing foam cell accumulation and inflammation *in vitro*. Then, the therapeutic effects of QA and EA (i.p., 5 mg/kg) were evaluated in ApoE knockout (ApoE^-/-^) mice fed a high-fat/high-cholesterol diet.

**Results:**

*In vitro* experiments demonstrated that both QA and EA suppressed oxidized low-density lipoprotein (ox-LDL)-induced foam cell formation by inhibiting cholesterol uptake and also inhibited lipopolysaccharide (LPS)-induced inflammatory responses. In high-fat/high-cholesterol diet-fed ApoE^-/-^ mice, QA or EA treatment significantly reduced serum lipid levels, decreased inflammatory factor release, and attenuated atherosclerotic plaque formation. At the tested dose, EA showed slightly better anti-inflammatory and plaque-reducing effects than QA. Transcriptomic profiling revealed enrichment of genes associated with “Cell adhesion molecules” and immune response pathways in the aorta of QA-treated mice. For EA, enrichment was observed for genes involved in “Circadian rhythms” in both liver and aorta, as well as genes related to “Vascular smooth muscle contraction” and the “cyclic guanosine monophosphate (cGMP)-protein kinase G (PKG) signaling pathway” in aortic tissues. Real-time quantitative PCR (qPCR) analysis confirmed altered expression of selected circadian rhythm-related genes in the liver of EA- and QA-treated mice and in ox-LDL-induced foam cells. EA also increased cGMP levels in these foam cells.

**Conclusions:**

This study provides the first evidence for the anti-atherosclerotic effects of QA and EA and offers new insights into the potential of natural products for the treatment of cardiovascular diseases.

## Introduction

Atherosclerosis (AS) is a chronic inflammatory arterial disease and a primary underlying cause of cardiovascular diseases (CVDs) and stroke (Bjoerkegren & Lusis, 2022). The characteristic feature of AS is the formation of plaques containing lipid, connective tissue and immune cells in the intima of large and medium-sized arteries (Engelen et al., 2022). The current clinical management of AS primarily involves lipid-lowering agents (*e.g*., statins). Additionally, therapeutic strategies targeting inflammation and immune responses in AS have shown promising progress (Engelen et al., 2022; Kong et al., 2022). However, some patients experience drug intolerance, drug resistance, or residual cardiovascular risks (*e.g*., residual lipid- and inflammation-associated risks) (Mach et al., 2024; Makover et al., 2023). Consequently, the discovery of highly effective and low-toxicity anti-atherosclerotic drug candidates from natural products has become an active area of research in recent years.

Quillaic acid (QA) and echinocystic acid (EA) are natural pentacyclic triterpenes (Fig. 1A) abundant in various herbs and have been reported to possess diverse pharmacological activities. QA, the primary aglycone of triterpene saponins found in Quillaja trees (*Quillaja saponaria* Mol.), exhibits a range of pharmacological activities, including anti-inflammatory, anticancer, antinociceptive and antihyperalgesic effects (Arrau et al., 2019; Huang et al., 2022). Notably, the inhibitory effects of QA and its derivatives on key inflammatory signaling pathways, such as nuclear factor kappa B (NF-κB) and mitogen-activated protein kinase (MAPK), suggest their potential for targeting the inflammatory processes in AS (Huang et al., 2022). EA is abundant in herbs such as *Eclipta prostrata* L. (Asteraceae) and *Albizzia julibrissin Durazz* (Fabaceae), and demonstrates a broad spectrum of pharmacological effects, including anti-inflammatory, antioxidant, neuroprotective, anticancer, anti-obesity, and anti-fatty liver activities (He et al., 2022; Kang et al., 2022; Xiao et al., 2023; Zhu et al., 2023). Several studies have indicated that EA possesses hypolipidemic activity (Han, Lai & Du, 2014), and can ameliorate hyperhomocysteinemia and oxidized low-density lipoprotein (ox-LDL)-induced vascular endothelial cell injury (Huang et al., 2017; Lai & Liu, 2014).

**Figure 1:**
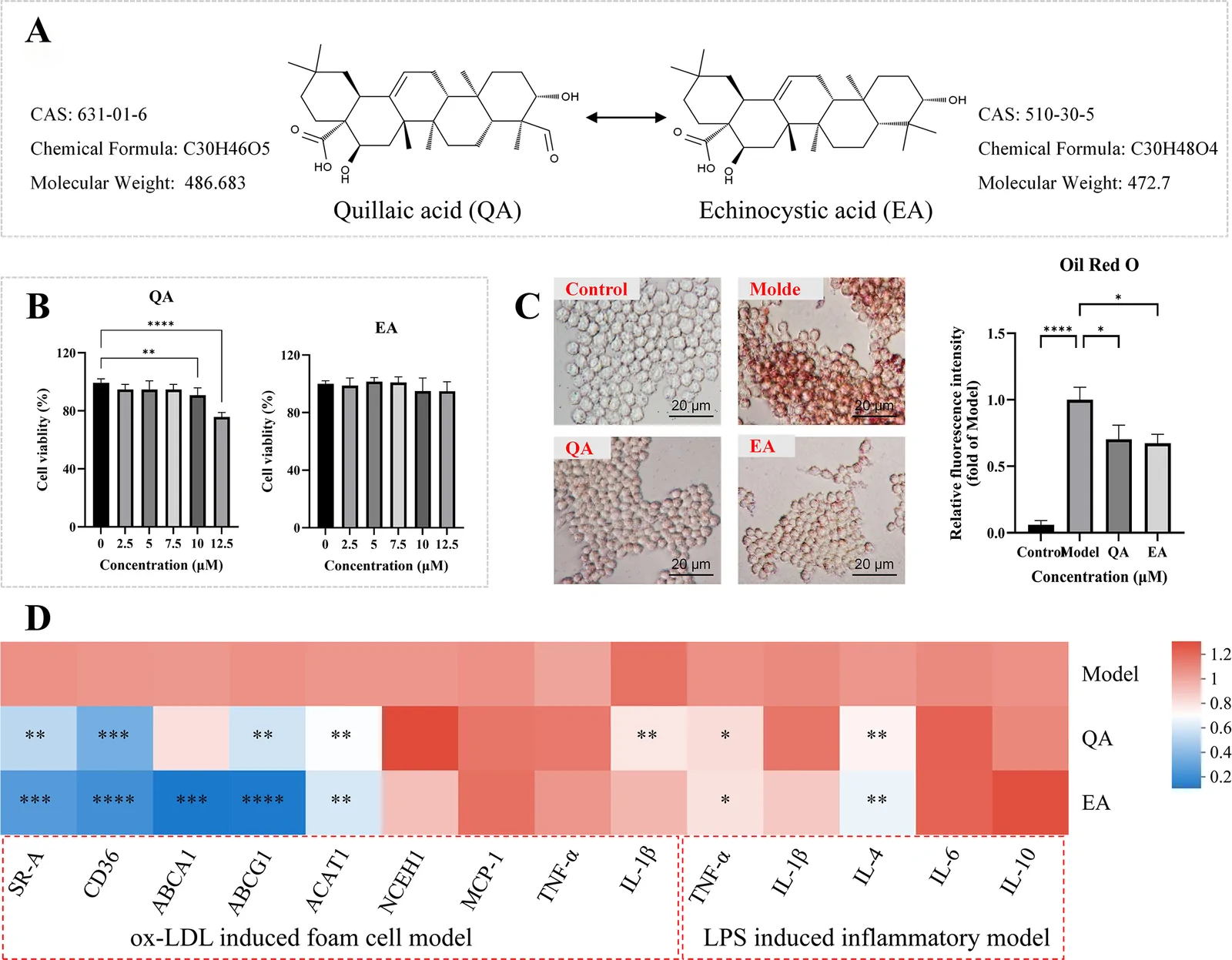
(A) Structure of QA and EA, (B) CCK-8 assay (OD 450 nm), (C) cellular oil red O staining, (D) qPCR analysis of key genes involved in foam cell formation and inflammation (*n* = 3 independent replicates). *p < 0.05, **p < 0.01, ***p < 0.001, and ****p < 0.0001.

However, the potential effects of QA and EA on ameliorating AS have not been investigated. This study was designed to systematically investigate and compare the effects of QA and EA on AS using a combination of *in vitro* models (*e.g*., foam cell formation and inflammation) and an *in vivo* ApoE knockout (ApoE^-/-^) mouse model of AS.

## Materials and Methods

### Cell culture and treatment

RAW 264.7 cells (ATCC, Manassas, VA, USA) were cultured in Dulbecco’s Modified Eagle’s Medium (DMEM) supplemented with 10% fetal bovine serum (FBS), 1% penicillin, and 1% streptomycin in an incubator (37 °C, 5% CO_2_). Cells (1 × 10^6^ cells per well) were seeded into 12-well plates and cultured for 6–8 h. Subsequently, the culture medium was replaced with fresh medium containing either QA or EA (purity >98%; BioBioPha, Yunnan, China) along with the modeling agents ox-LDL (50 μg/mL, #IO1300) or lipopolysaccharide (LPS) (1 μg/mL, #IL2020), both purchased from Solarbio Science & Technology Co., Ltd. (Beijing, China). After 24 h of treatment, the cells were harvested for subsequent experiments. All cell-based experiments were performed in three independent replicates (*n* = 3).

### CCK-8 assay

Cells were seeded in 96-well plates at a density of 5 × 10^4^ cells per well and allowed to adhere for 6–8 h in an incubator. After cell attachment, the culture medium was replaced with fresh medium containing various concentrations (0–12.5 μM) of QA, EA, or the solvent control dimethyl sulfoxide (DMSO, final concentration below 0.1%). Following a 24 h incubation period, cell viability was assessed using a CCK-8 kit (#A311; Vazyme Biotech Co., Ltd., Nanjing, China) according to the manufacturer’s instructions.

### Cellular oil red O staining

After the indicated treatments, intracellular lipid droplets were stained using an Oil Red O Staining Kit (#G1262; Solarbio Science & Technology Co., Ltd., Beijing, China) according to the manufacturer’s instructions. Lipid droplets were quantitatively analyzed using Image-Pro Plus software.

### Enzyme-linked immunosorbent assay

After ox-LDL stimulation and QA/EA treatment, the cell supernatant was removed, and the cells were lysed in RIPA buffer containing protease inhibitors (#P0038; Beyotime Biotechnology Co., Ltd., Shanghai, China). The intracellular cyclic guanosine monophosphate (cGMP) concentration in the lysate was then determined using an Enzyme-linked immunosorbent assay (ELISA) kit (#ml001887; Shanghai Enzyme-linked Biotechnology Co., Ltd., Shanghai, China) according to the manufacturer’s instructions.

### Animal experiment design

Seven-week-old male ApoE^-/-^ mice, weighing 20.5 ± 1.5 g, were purchased from Gempharmatech Co., Ltd. (Jiangsu, China). The mice were housed in standard cages, with four mice per cage, and maintained in a controlled condition (12/12 h light/dark, 25 ± 2 °C, 40–70% humidity) with *ad libitum* access to food and water. The study protocol was approved by the Ethics Committee of Jiangnan University (JN.No20241030t1100214[553]), and the study strictly adhered to institutional guidelines for animal welfare.

After a 1-week acclimation, mice were randomly assigned to four groups (eight mice per group): the Control group, the Model group, the QA group, and the EA group. The Model, QA, and EA groups were fed a high-fat/high-cholesterol diet (#XT108C), and the Control group received a chow diet (#XT1082C). All diets were purchased from Jiangsu Xietong Pharmaceutical Bio-engineering Co., Ltd. (Nanjing, China). QA and EA (5 mg/kg) were dissolved in a vehicle of 5% DMSO in saline and administered *via* intraperitoneal injection five times per week for 12 weeks. The single 5 mg/kg dose was selected to allow a fair direct comparison under identical conditions, based on previous *in vivo* studies of EA at this dose (He et al., 2022), the structural similarity between QA and EA, and the exploratory nature of this first investigation of their anti-atherosclerotic effects. The Control and Model groups received vehicle injections on the same schedule.

At the end of the experiment, all mice were euthanized by cervical dislocation after being anesthetized with isoflurane. The detailed procedure was as follows: the experimenter grasped the base of the tail, lifted the mouse, placed it on the cage lid, and pressed firmly on the head and neck with the thumb and index finger of the other hand while pulling the tail backward and upward to cause dislocation of the cervical spine and separation of the spinal cord from the brain stem. The animals died immediately. Serum, liver, heart, and aorta samples were collected as described by Han et al. (2022).

### Biochemical and luminex analysis

Following collection from the retro-orbital plexus, blood samples were kept at room temperature for 1 h to permit complete clotting. Subsequently, the samples were centrifuged at 3,000× *g* for 15 min to obtain serum. The concentrations of triglyceride (TG), total cholesterol (TC), high-density lipoprotein cholesterol (HDL-C), and low-density lipoprotein cholesterol (LDL-C) in the serum were quantified with a Mindray BS-480 automated biochemical analyzer (Mindray Bio-Medical Electronics Co., Ltd., Shenzhen, China). Mouse serum samples were thawed and analyzed by Luminex multiplex cytokine assay according to the manufacturer’s instructions (LabEx, Shanghai, China). Cytokines measured included tumor necrosis factor-α (TNF-α), monocyte chemoattractant protein-1 (MCP-1), interleukin (IL)-1β, IL-4, IL-6, IL-10, IL-12p70, and macrophage colony-stimulating factor (M-CSF).

### Histopathology and immunofluorescence analysis

Atherosclerotic lesions in the aortic arch were assessed by Oil Red O staining to visualize lipid deposition. The aortic root was embedded in optimal cutting temperature (OCT) compound, and the aortic sinus was sectioned into serial 7-µm-thick slices. Histopathological evaluation was performed using hematoxylin and eosin (H&E) staining and Masson staining. Immunofluorescence staining was used to measure the expression of F4/80 (a macrophage marker) and α-SMA (a smooth muscle cell marker). Histopathological analysis was performed by Servicebio Technology Co., Ltd. (Wuhan, China). All images were acquired using a Pannoramic MIDI (3DHISTECH, Budapest, Hungary) and analyzed with ImageJ and Image-Pro Plus software.

### RNA sequencing analysis

RNA sequencing (RNA-seq) analysis was performed using previously published methods (Zhong et al., 2023). Briefly, total RNA was extracted from frozen livers and aortas (10–20 mg) using TRIzol® Reagent according to the manufacturer’s instructions. RNA-seq was performed with five biological replicates per group, each replicate derived from an individual mouse. Then, RNA quality was determined by a 5300 Bioanalyser (Agilent, Santa Clara, CA, USA) and quantified using the ND-2000 (NanoDrop Technologies, Wilmington, DE, USA). High-quality RNA samples were used to construct the sequencing library. Each library was sequenced once (no technical replicates). RNA-seq was performed by Majorbio Bio-Pharm Technology Co., Ltd. (Shanghai, China). RNA-seq results were processed on the online MajorBio Cloud Platform (www.majorbio.com). The thresholds for determining differentially expressed genes (DEGs) were as follows: *P*adjust < 0.05, |Fold change| > 2. The statistical power of this experimental design, calculated in the R package RNASeqPower, is 67–83% for detecting two-fold changes (two-sided α = 0.05; coefficient of variation 0.3–0.4).

### Real-time quantitative PCR

Total RNA was extracted from cells (*n* = 3 independent replicates) or liver tissues (*n* = 5 mice per group) using an RNA extraction kit (#RC112), and then was reverse transcribed into cDNA according to the manufacturer’s instructions (#R333). Then, Real-time quantitative PCR (qPCR) was performed using ChamQ Blue Universal SYBR qPCR Master Mix (#Q312) according to the manufacturer’s protocol. These kits were purchased from Vazyme Biotech Co., Ltd. (Nanjing, China). Finally, the mRNA levels were evaluated relative to β-actin using the 2^−ΔΔCt^ method. The primers used in the experiment are presented in Table S1.

### Statistical analysis

All data are presented as the mean ± standard deviation (SD). Data analysis and graphical presentation were performed using GraphPad Prism 9 software (GraphPad Software, Inc., San Diego, CA, USA). The statistical significance was assessed using one-way analysis of variance (ANOVA), followed by Tukey’s *post-hoc* test for intergroup comparisons. Differences were considered statistically significant at *p < 0.05, **p < 0.01, ***p < 0.001, or ****p < 0.0001.

## Results

### QA and EA inhibit foam cell formation and inflammation

CCK-8 results showed that QA was cytotoxic at concentrations ≥10 μM, while EA was not cytotoxic up to 12.5 μM (Fig. 1B), leading to the use of 7.5 μM for both QA and EA in subsequent cell experiments. After 24 h of ox-LDL stimulation of RAW 264.7 macrophages, Oil Red O staining showed that cells in the Model group exhibited abundant red lipid droplets, indicating foam cell formation. The area of red lipid droplets was significantly reduced in the QA (*p* < 0.05) and EA (*p* < 0.05) groups (Fig. 1C). As shown in Fig. 1D, after 24 h of ox-LDL stimulation in RAW 264.7 cells, QA and EA significantly reduced the expression of the genes *SR-A*, *CD36*, *ABCG1* and *ACAT1* (*p* < 0.01 to *p* < 0.0001), and only EA significantly reduced *ABCA1* expression (*p* < 0.001). Neither QA nor EA significantly affected *NCEH1* expression. Furthermore, QA significantly suppressed ox-LDL-induced *IL-1β* expression (*p* < 0.01), while neither QA nor EA affected the expression of *MCP-1* and *TNF-α* (Fig. 1D). Additionally, following LPS-induced cellular inflammation, QA and EA significantly suppressed the expression of the inflammatory mediators *TNF-α* (*p* < 0.05) and *IL-4* (*p* < 0.01), while they did not significantly affect *IL-1β*, *IL-6* or *IL-10* expression (Fig. 1D). These findings indicate that QA and EA effectively inhibit foam cell formation and alleviate specific aspects of cellular inflammation.

### QA and EA improve serum lipid profile and inflammation in ApoE^-/-^ mice

An AS model was established in ApoE^-/-^ mice using a high-fat/high-cholesterol diet, with QA and EA administered *via* intraperitoneal injection (Fig. 2A). Body weight measurements revealed that the high-fat/high-cholesterol diet and drug interventions had no significant impact on the mice’s growth (Figs. 2B and 2C). As expected, the high-fat/high-cholesterol diet significantly increased serum TC (*p* < 0.0001), TG (*p* < 0.05) and LDL-C (*p* < 0.0001) levels compared with the control diet, while HDL-C remained unchanged (Figs. 2D–2G). After the 12-week intervention, both QA and EA treatments significantly attenuated the high-fat/high-cholesterol diet-induced dyslipidemia, as evidenced by reduced serum TC (*p* < 0.01 for QA; *p* < 0.05 for EA) and LDL-C (*p* < 0.05 for both). Only QA significantly decreased serum HDL-C levels (*p* < 0.05). The high-fat/high-cholesterol diet also induced a systemic inflammatory response, significantly elevating serum levels of TNF-α (*p* < 0.001), IL-6 (*p* < 0.05), and MCP-1 (*p* < 0.05) compared with the Control group, without significantly altering IL-1β, IL-4, IL-10, IL-12p70, or M-CSF (Figs. 2H–2O). Compared with the Model group, both QA and EA treatments significantly reduced serum TNF-α levels (*p* < 0.01), and only EA specifically reduced serum levels of IL-6 (*p* < 0.05) and MCP-1 (*p* < 0.05). In addition, EA significantly reduced serum IL-4 levels (*p* < 0.05), while QA significantly decreased serum IL-12p70 levels (*p* < 0.05). In summary, these findings demonstrate that both QA and EA can ameliorate high-fat/high-cholesterol diet-induced lipid metabolic disorders and inflammation, thereby exerting protective effects against AS in mice. Notably, EA appears to exhibit a slightly superior anti-inflammatory profile compared with QA.

**Figure 2:**
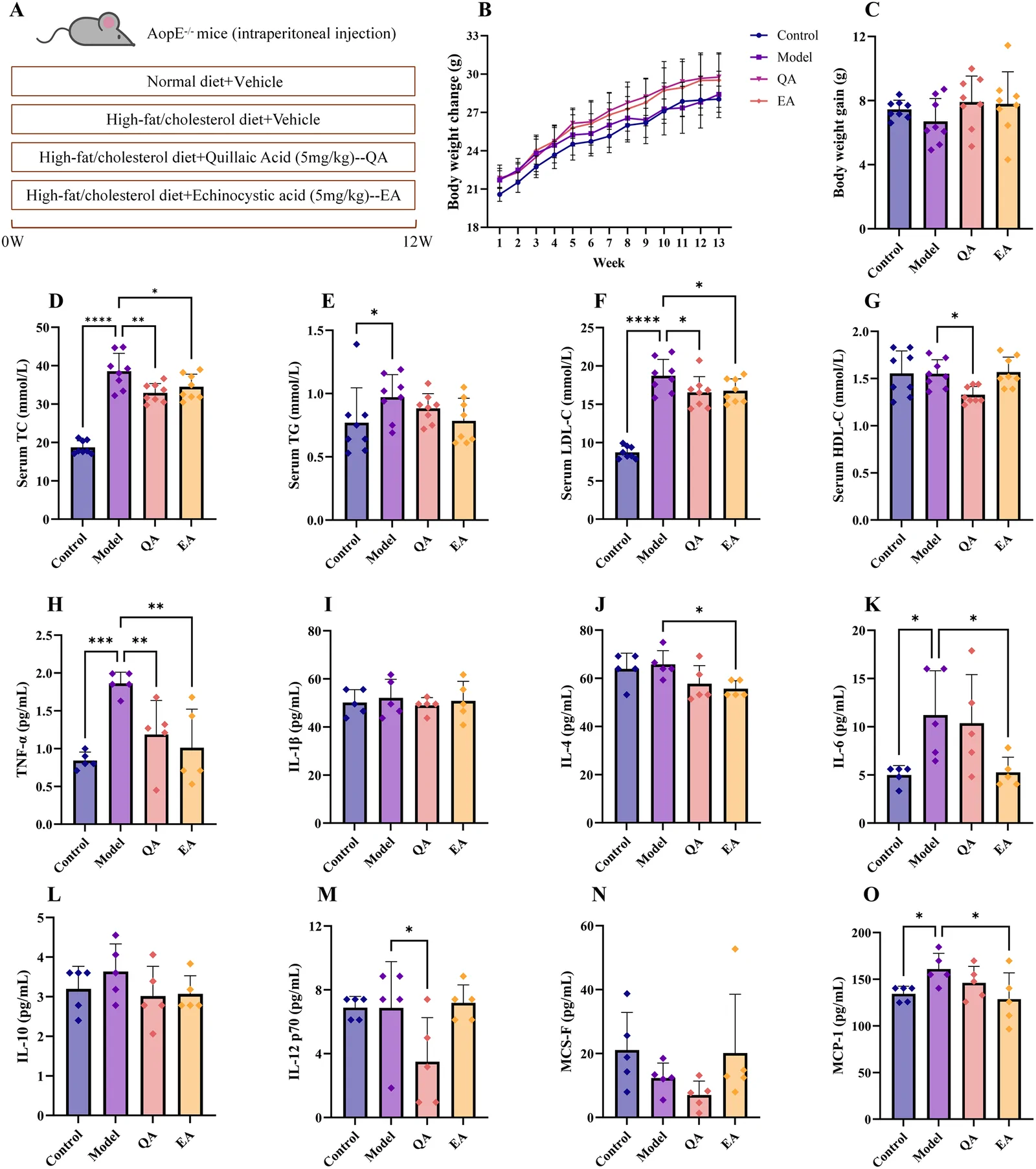
(A) Animal experiment design, (B, C) body weight changes and body weight gain, (D–G) serum levels of TC, TG, LDL-C and HDL-C, (H–O) serum levels inflammatory cytokines (*n* = 5–8 mice per group). *p < 0.05, **p < 0.01, ***p < 0.001, and ****p < 0.0001.

### QA and EA inhibit atherosclerotic plaque formation in ApoE^-/-^ mice

The impact of QA and EA on atherosclerotic plaque development was evaluated by Oil Red O staining in ApoE^-/-^ mice. As shown in Fig. 3A, the Model group exhibited a larger plaque area (stained in red) compared with the Control group, confirming the successful induction of AS by the high-fat/high-cholesterol diet. Treatment with either QA or EA significantly inhibited plaque formation. Histopathological analysis by H&E staining revealed that the Control group maintained intact aortic root structures with only minor pathological changes (Fig. 3B). In contrast, the Model group displayed severe pathological alterations, including proliferative changes in the aortic intima, luminal narrowing, substantial intimal thickening, and extensive inflammatory cell infiltration (Fig. 3B). Quantification revealed a significantly larger plaque area in the Model group than in the Control group (Fig. 3D). Both the QA and EA groups showed a marked improvement, with significantly reduced plaque area and attenuated pathological damage compared with the Model group, although intimal thickening and inflammatory infiltration were still present (Figs. 3B and 3D). Masson staining revealed increased collagen deposition (blue) in the Model, QA, and EA groups, indicating fibrous cap formation (Fig. 3C). However, quantitative analysis showed that neither QA nor EA treatment significantly altered the collagen content within plaques compared with the Model group (Fig. 3E). These results indicate that both QA and EA can inhibit atherosclerotic plaque formation in ApoE^-/-^ mice, but neither demonstrates significant effects on structural repair or stabilization of arterial plaques.

**Figure 3:**
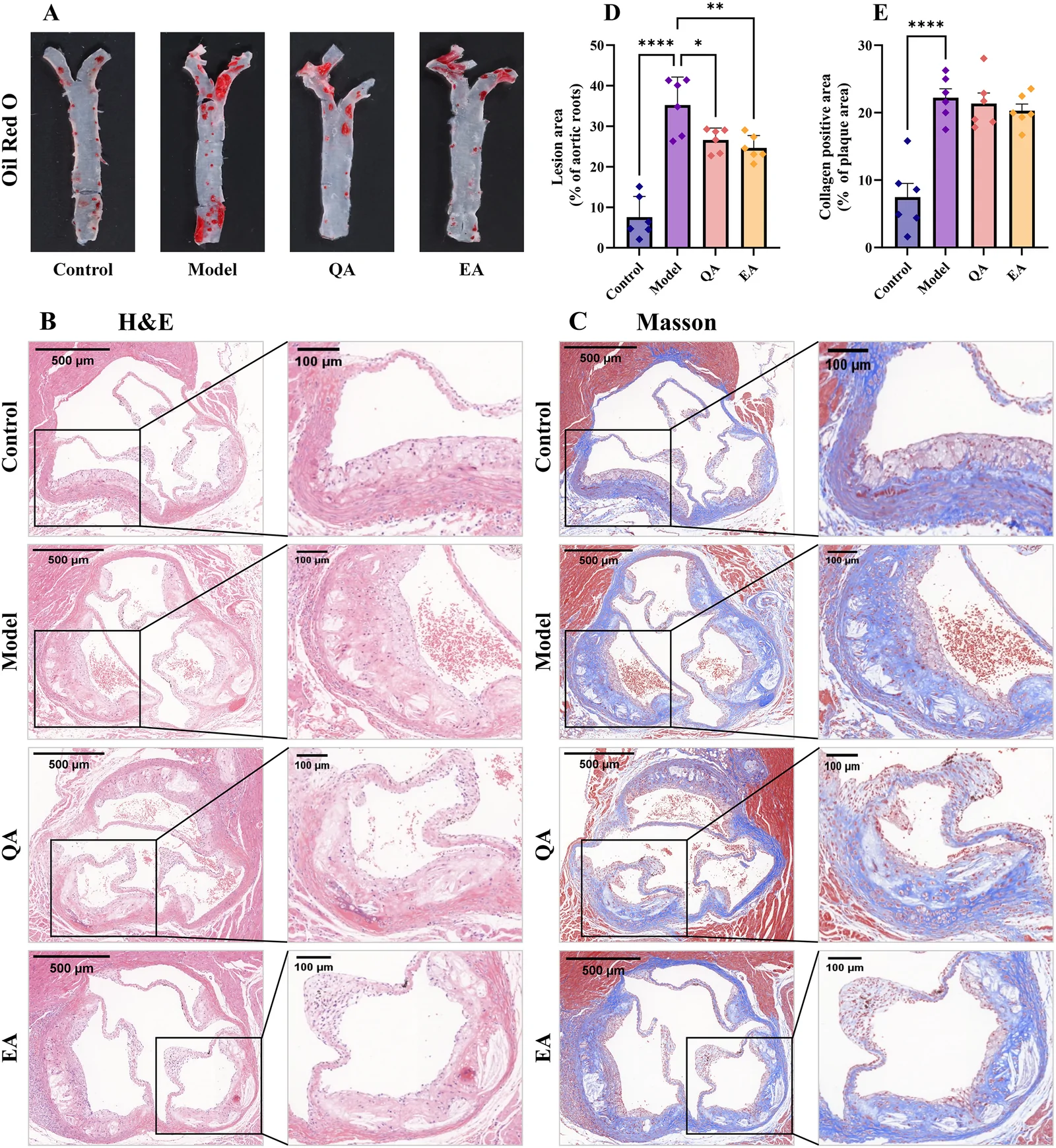
(A) Oil red O staining of mouse aortic arch, (B, C) H&E and masson staining of mouse aortic root, (D) plaque area at the root of the mouse aorta, (E) collagen content in plaques at the root of the mouse aorta (*n* = 6 mice per group). *p < 0.05, **p < 0.01, and ****p < 0.0001.

### Effects of QA and EA on the expression of F4/80 and α-SMA

In this study, immunofluorescence staining was used to perform the localization and semi-quantitative analysis of the macrophage marker F4/80 and the smooth muscle cell marker α-SMA. In the Control group, plaques exhibited weaker F4/80 (red) and stronger α-SMA (green) fluorescence signals, indicating low macrophage presence and relatively preserved smooth muscle cell (SMC) content (Fig. 4). In contrast, the Model group displayed a significant increase in F4/80 expression (*p* < 0.01) and a marked decrease in α-SMA expression (*p* < 0.05). Treatment with either QA or EA significantly ameliorated these changes, as evidenced by a notable reduction in F4/80 expression (*p* < 0.05) and a concurrent increase in α-SMA expression (*p* < 0.05) compared with the Model group (Fig. 4). These findings suggest that QA and EA may alleviate AS by reducing macrophage infiltration, and promoting smooth muscle cell proliferation and migration.

**Figure 4:**
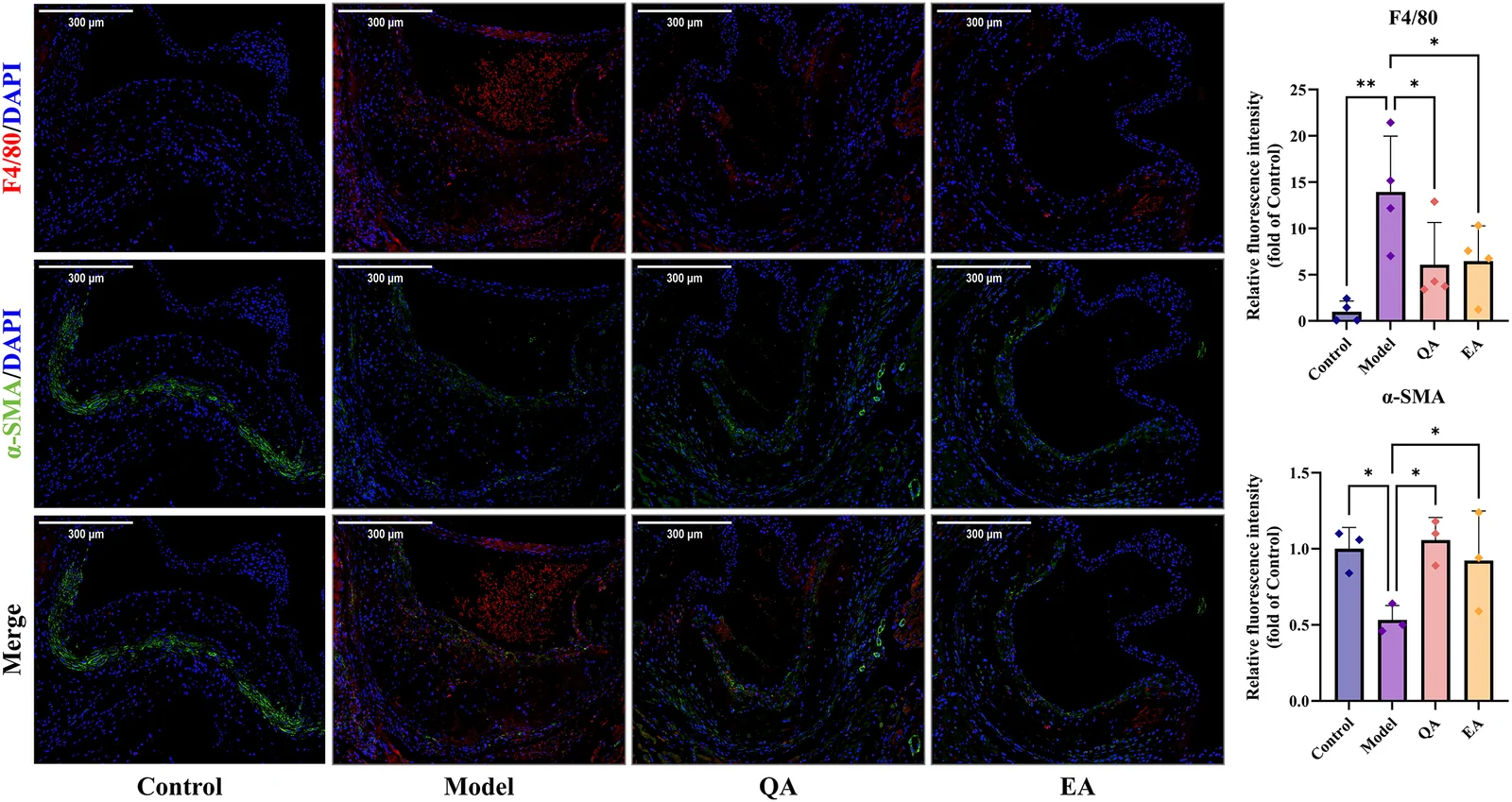
Immunofluorescence staining for F4/80 and α-SMA in the aortic root of mice (*n* = 3–4 mice per group). *p < 0.05 and **p < 0.01.

### QA and EA modified gene expression profiles of liver

To elucidate the potential molecular mechanisms of QA and EA in the liver, we conducted a transcriptomic analysis of liver tissues from ApoE^-/-^ mice. The clean data for each sample reached more than 6 Gb, with a Q30 base percentage above 96.63%. The clean reads from each sample were aligned to the designated reference genome, yielding alignment rates ranging from 95.11% to 97.95%. Principal component analysis (PCA) showed distinct clustering of gene expression profiles among the Model, QA, and EA groups (Fig. 5A). Differential expression analysis identified 40 genes (28 upregulated, 12 downregulated) in the QA group and 2,502 genes (2,050 upregulated, 452 downregulated) in the EA group compared with the Model group, indicating a more profound transcriptional impact of EA (Fig. 5B). A Venn diagram revealed 15 DEGs common to both treatment groups (Fig. 5C). Gene Ontology (GO) enrichment analysis of the top 15 terms showed that QA-regulated DEGs were significantly associated with 106 terms (*P*adjust < 0.05), primarily related to biological processes including “protein-hormone receptor activity”, “negative regulation of gluconeogenesis” and “insulin receptor activity”, *etc*. (Fig. 5D). In contrast, EA-regulated DEGs were enriched in 71 GO terms (*P*adjust < 0.05), predominantly involving molecular functions such as “metal ion binding”, “cation binding” and “ion binding”, and biological processes including “regulation of nucleobase-containing compound metabolic process”, “regulation of primary metabolic process” and “regulation of RNA metabolic process” (Fig. 5D). Kyoto Encyclopedia of Genes and Genomes (KEGG) pathway analysis indicated that DEGs in the QA group were not significantly enriched in any specific pathway (*P*adjust > 0.05), whereas DEGs in the EA group were significantly enriched in pathways including “Herpes simplex virus 1 infection” and “Circadian rhythm” (*P*adjust < 0.05). Previous studies have shown that feeding ApoE^-/-^ mice a high-fat/high-cholesterol diet leads to an imbalance in circadian rhythm-related gene expression (Casagrande et al., 2025). We further analyzed the DEGs in the “Circadian rhythm” pathway. As detailed in Table 1, EA significantly upregulated the expression of *Prkag3*, *Nr1d2*, *Creb1*, *Ptp4a1*, *Per3*, *Bhlhe40*, *Bhlhe41*, *Per2*, and *Dbp* (*P*adjust < 0.05), while significantly downregulating *Npas2*, *Bmal1* and *Nfil3* (*P*adjust < 0.05). As shown in Fig. 5F, further qPCR validation of a subset of genes associated with the “Circadian rhythm” revealed that compared with the Control group, the Model group had significantly higher hepatic *Dbp* and *Per3* expression and lower *Nr1d2* expression (*p* < 0.01 to *p* < 0.0001). Compared with the Model group, QA significantly upregulated the expression of *Dbp* (*p* < 0.05) and *Per2* (*p* < 0.0001) in the liver. Additionally, compared with the Model group, EA significantly upregulated the expression of *Dbp*, *Per2*, *Per3*, and *Nr1d2* in the liver and significantly downregulated *Npas2* expression (*p* < 0.05 to *p* < 0.0001). These results were largely consistent with the RNA sequencing data.

**Figure 5:**
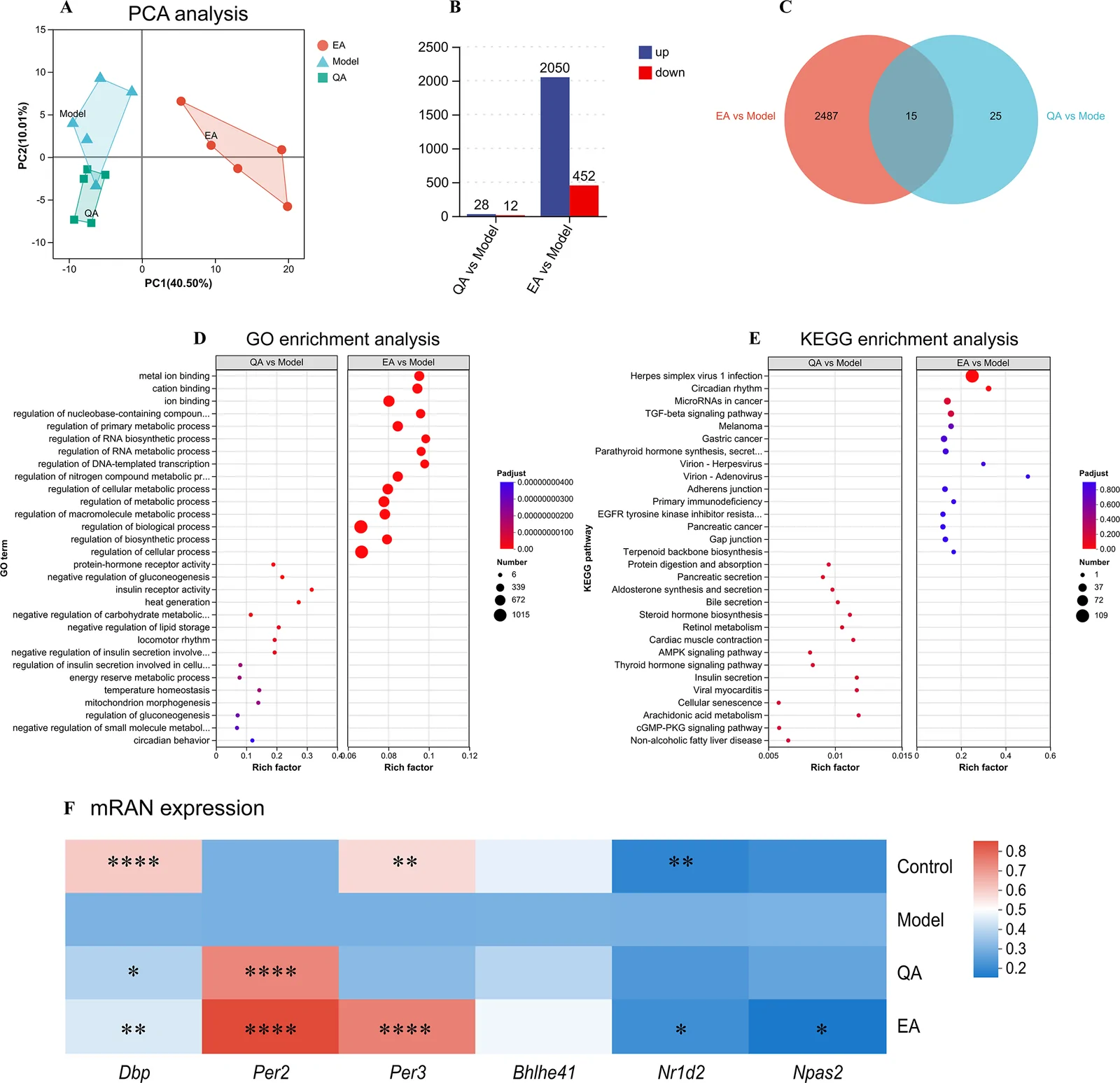
Transcriptome profiles in livers of different groups. (A) PCA analysis, (B) differential statistics, (C) venn analysis of DEGs, (D) GO enrichment analysis, (E) KEGG enrichment analysis, (F) the expression of genes related to “Circadian rhythm” in the liver. *p < 0.05, **p < 0.01, and ****p < 0.0001.

**Table 1:** Key KEGG pathways (*P*adjust < 0.05) and associated DEGs in atherosclerosis.

Group	Pathway description	Down-regulated	Up-regulated
EA *vs*. Model (liver)	Circadian rhythm	*Npas2, Bmal1, Nfil3*	*Prkag3, Nr1d2, Creb1, Ptp4a1, Per3, Bhlhe40, Bhlhe41, Per2, Dbp*
QA *vs*. Model (aorta)	Cell adhesion molecules	*H2-DMa, Itgal, Ctla4, H2-DMb2, H2-Oa, H2-Eb2, Cd2, Sell, Glycam1, H2-Q6, Itgb7, H2-Ab1, H2-Ob, H2-Q7, Cd6*	–
	Th17 cell differentiation	*H2-DMa, H2-DMb2, H2-Oa, Zap70, Il2rb, Cd3d, Cd3e, H2-Ab1, Foxp3, H2-Ob, H2-Eb2*	–
	Th1 and Th2 cell differentiation	*H2-DMa, H2-DMb2, H2-Oa, Zap70, Il2rb, Cd3d, Cd3e, H2-Ab1, H2-Ob, H2-Eb2*	–
	Antigen processing and presentation	*H2-DMa, H2-DMb2, H2-Oa, H2-Eb2, Tap1, H2-Q6, H2-Ab1, H2-Ob, H2-Q7*	–
EA *vs*. Model (aorta)	Vascular smooth muscle contraction	*Cacna1s, Prkcq, Kcnu1, Myl6b*	*Braf, Myh11, Ppp1r12a, Rock2, Mylk, Rock1, Cald1, Pla2g4c, Mylk4, Plcb1, Prkg1*
	Circadian rhythm	*Npas2, Nfil3*	*Nr1d2, Per3, Bhlhe41, Per2, Dbp*
	cGMP-PKG signaling pathway	*Cacna1s, Kcnu1, Adrb3, Fxyd2*	*Ppp1r12a, Rock2, Mylk, Rock1, Mylk4, Plcb1, Prkg1, Prkg2, Pde3a, Myh7, Pde5a*

### QA and EA modified gene expression profiles of aorta

Transcriptomic analysis was subsequently performed on aortic tissues from ApoE^-/-^ mice. The clean data for each sample reached more than 5.88 Gb, with a Q30 base percentage above 95.24%. The clean reads from each sample were aligned to the designated reference genome, yielding alignment rates ranging from 97.56% to 98.69%. PCA of aortic gene expression profiles showed clear separation among the Model and intervention groups, with partial overlap between the two treatment groups (Fig. 6A). Differential expression analysis identified 176 DEGs (34 upregulated, 142 downregulated) in the QA group and 833 DEGs (572 upregulated, 261 downregulated) in the EA group compared with the Model group, suggesting a more extensive transcriptional impact of EA (Fig. 6B). A total of 87 DEGs were shared between the QA and EA treatment groups (Fig. 6C). GO enrichment analysis (Fig. 6D) of the top 15 terms showed that QA-regulated DEGs were significantly associated with immune-related processes, including “immune system process”, “immune response” and “regulation of immune system process”. In contrast, EA-regulated DEGs were enriched in broader regulatory processes including “regulation of cellular process”, “biological regulation” and “regulation of biological process”. KEGG pathway analysis (Fig. 6E) further showed distinct transcriptional profiles. QA-regulated DEGs were significantly enriched in pathways related to immune regulation, such as “Hematopoietic cell lineage”, “Cell adhesion molecules”, “Epstein-Barr virus infection”, “Th17 cell differentiation”, “Th1 and Th2 cell differentiation” and “Antigen processing and presentation” (*P*adjust < 0.05). Conversely, EA-regulated DEGs were significantly enriched in four pathways, namely “Herpes simplex virus 1 infection”, “Vascular smooth muscle contraction”, “Circadian rhythm” and “cGMP-PKG signaling pathway” (*P*adjust < 0.05). A detailed examination of DEGs within AS-relevant pathways (Table 1) confirmed that QA consistently downregulated genes in the signaling pathways of “Cell adhesion molecules”, “Th17 cell differentiation”, “Th1 and Th2 cell differentiation” and “Antigen processing and presentation”. Meanwhile, EA-regulated DEGs were predominantly enriched in the “Vascular smooth muscle contraction”, “Circadian rhythm”, and “cGMP-PKG signaling” pathways, suggesting a distinct transcriptional profile related to vascular physiology. The “Circadian rhythm” pathway was also significantly enriched in the KEGG analysis of the liver transcriptome; hence, an ox-LDL-induced foam cell model was established to validate its key genes using qPCR. As shown in Fig. 6F, compared with the Control group, the Model group had significantly higher *Dbp* and *Per3* expression and lower *Bhlhe41* expression (*p* < 0.05 to *p* < 0.01). Compared with the Model group, QA significantly upregulated the expression of *Per3* and *Bhlhe41* (*p* < 0.05 to *p* < 0.01). Additionally, compared with the Model group, EA significantly upregulated the expression of *Dbp*, *Per2*, *Per3*, *Bhlhe41* and *Nr1d2* (*p* < 0.05 to *p* < 0.001), and significantly downregulated the expression of *Npas2* (*p* < 0.01). Furthermore, in the ox-LDL-induced foam cell model (Fig. 6G), QA did not significantly alter cGMP levels, whereas EA significantly increased cellular cGMP levels (*p* < 0.01), suggesting that EA may activate the cGMP-PKG signaling pathway.

**Figure 6:**
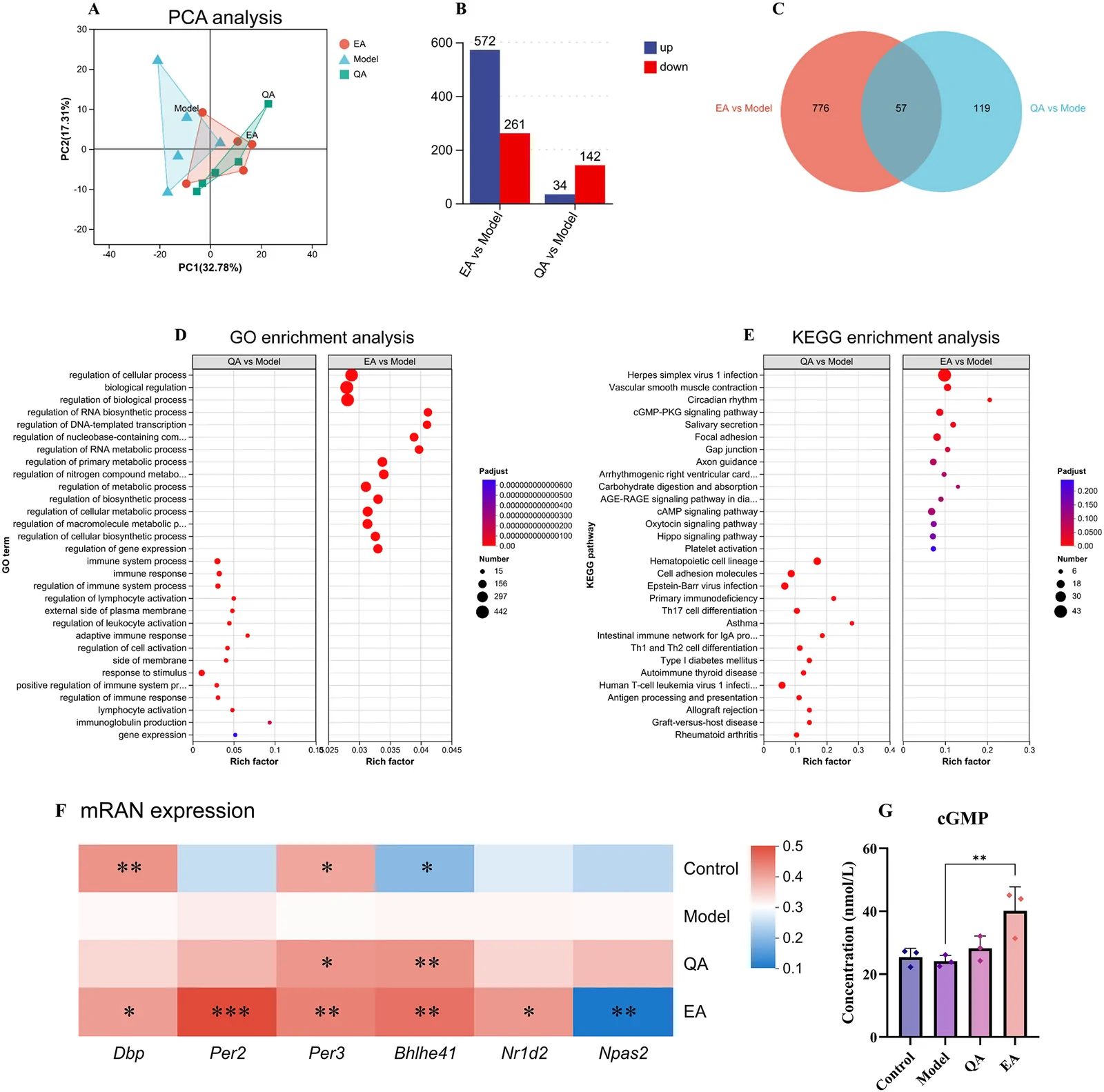
Transcriptome profiles in aortas of different groups. (A) PCA analysis, (B) differential statistics, (C) venn analysis of DEGs, (D) GO enrichment analysis, (E) KEGG enrichment analysis, (F) the expression of genes related to “Circadian rhythm” in foam cells, (G) cGMP levels in foam cells. *p < 0.05, **p < 0.01, and ***p < 0.001.

## Discussion

The bark of *Quillaja saponaria* Mol. contains high concentrations of triterpene saponins, including QA, which have been used for centuries in Chilean folk medicine as cleansing, anti-inflammatory, and analgesic agents (Arrau et al., 2019). However, limited information is available on the metabolic stability and bioavailability of QA. In contrast, the safety of EA has been widely demonstrated, and EA has been reported as an ingredient in foods and traditional Chinese medicine in many Asian countries (Chen et al., 2020). Some studies suggest that orally administered lancemaside A may be converted to EA by the gut microbiota, and that EA is then absorbed into the bloodstream, where it exerts anti-inflammatory effects and ameliorates scopolamine-induced memory and learning deficits (Joh & Kim, 2010; Jung et al., 2012). Notably, the KEGG database (https://www.kegg.jp/) shows that EA can be metabolically converted to QA *via* the enzymatic action of CYP714E52.

Numerous natural products have been shown to ameliorate AS by modulating lipid metabolism, suppressing inflammatory responses, or inhibiting foam cell formation (Xiaobei et al., 2022; Zhao et al., 2021). However, whether QA and EA exert similar anti-atherosclerotic effects remains unknown. In this study, we employed ox-LDL and LPS to stimulate RAW 264.7 macrophages *in vitro*, establishing foam cell and inflammatory models, respectively, to preliminarily evaluate the anti-atherosclerotic potential of QA and EA. Foam cells are central to the development of atherosclerotic plaques, with their formation governed by processes including cholesterol uptake, esterification, and efflux (Gui, Zheng & Cao, 2022; Wang et al., 2019). Key strategies to inhibit foam cell formation involve reducing cholesterol uptake, particularly *via* scavenger receptors CD36 and SR-A, which mediate 75–90% of modified lipoprotein uptake, as well as modulating esterification and promoting cholesterol efflux (Gui, Zheng & Cao, 2022; Wang et al., 2019). Moreover, inflammation plays a critical role in the pathophysiology of CVDs and is instrumental in the initiation and progression of atherosclerotic lesions (Engelen et al., 2022; Kong et al., 2022). Our findings indicate that QA and EA attenuate foam cell formation primarily by downregulating the expression of *SR-A* and *CD36*, thereby reducing cholesterol uptake in macrophages. Interestingly, however, QA and/or EA also suppressed the expression of genes involved in cholesterol esterification (*ACAT1*) and efflux (*ABCG1* and *ABCA1*) (Fig. 1D). Although this pattern may appear paradoxical at first glance, it is mechanistically coherent when interpreted as a feedback response to reduced cholesterol influx: because SR-A and CD36 mediate the majority (75–90%) of modified lipoprotein uptake, their downregulation by QA and EA reduces the intracellular cholesterol pool, and the downstream genes involved in esterification and efflux are correspondingly downregulated. Nevertheless, we acknowledge that intracellular TC and cholesteryl ester contents were not directly quantified, and direct biochemical quantification would further substantiate the net effect of QA and EA on the intracellular cholesterol balance; this will be addressed in future studies. Additionally, in LPS-induced inflammatory models, both QA and EA markedly inhibited the expression of key inflammatory mediators, including *TNF-α* and *IL-4*, underscoring their anti-inflammatory properties.

The Western-style diet, characterized by high fat and high cholesterol intake, has emerged as a key contributor to CVDs (Adolph & Tilg, 2024). In this study, ApoE^-/-^ mice were fed a high-fat/high-cholesterol diet, which induced hyperlipidemia, inflammation, and aortic plaque formation. Even in the Control group fed a standard chow diet, mild plaque accumulation was observed in the aortas of ApoE^-/-^ mice. This phenomenon can be attributed to the fact that ApoE^-/-^ mice on a regular diet accumulate VLDL remnant lipoproteins and chylomicrons, leading to human-like type III hyperlipidemia (Ilyas et al., 2022). Both QA and EA were found to ameliorate disorders in lipid metabolism, reduce inflammation, and attenuate plaque accumulation. F4/80, a well-established macrophage marker (Austyn & Gordon, 1981; Li et al., 2023b), showed increased expression indicative of enhanced macrophage infiltration within atherosclerotic plaques. Similarly, the distribution and number of α-SMA-positive cells (α-SMA is a vascular SMC-specific marker (Burger et al., 2022)) reflect the state of SMCs within plaques, and the proliferation and migration of these cells are crucial for plaque formation and stabilization. Both QA and EA significantly reduced the relative expression of F4/80 in the aortic root of mice (Fig. 4), indicating diminished macrophage infiltration. Specifically, considering the significant reduction in serum MCP-1 levels observed in the EA group, the decreased macrophage numbers may originate from blood monocytes, which can migrate into the intima, and differentiate into macrophages (Bjoerkegren & Lusis, 2022). In addition, QA and EA significantly increased the relative expression of α-SMA in the aortic root of mice (Fig. 4), indicating that they may promote SMC proliferation and migration, thereby facilitating plaque repair and stabilization (Basatemur et al., 2019).

Liver dysfunction is closely associated with the development and progression of AS (Castillo-Nunez et al., 2024). EA treatment was associated with more extensive transcriptional changes in the liver than QA, suggesting that the two compounds may affect distinct sets of genes. Functional enrichment analysis indicated that genes associated with hormone receptor activity and glucose metabolism were overrepresented in the QA group, suggesting a possible link to metabolic regulation. In the EA group, enrichment was observed for genes related to ion binding. Enrichment of genes related to circadian rhythm was also observed in the EA group. Circadian rhythms are important for vascular function, and their disruption has been associated with CVDs (Costello et al., 2023; Geng et al., 2023). Specifically, qPCR analysis revealed that, in both mouse livers and foam cells, *Npas2* gene expression was downregulated, while *Dbp*, *Per2*, *Per3* and *Nr1d2* gene expression were upregulated (Figs. 5F and 6F). GO and KEGG enrichment analyses showed enrichment of immune-related pathways in the QA group. During AS development, ox-LDL and inflammatory mediators stimulate endothelial cell activation to express adhesion molecules (*e.g*., P-selectin, E-selectin, VCAM1 and ICAM-1) (Bjoerkegren & Lusis, 2022). This process promotes the adhesion of monocytes, ultimately leading to the differentiation of monocytes into macrophages.

Genes related to “Cell adhesion molecules” were downregulated in the QA group (Table 1), consistent with the reduced expression of F4/80 and inflammatory mediators. T cell subsets and their functions are known to influence the development and progression of AS (Saigusa, Winkels & Ley, 2020). Genes related to T cell function were downregulated in the QA group (Table 1), suggesting a possible immunomodulatory component. In the EA group, differentially expressed genes were enriched for terms related to biological regulation. Previous studies have reported downregulation of genes related to “Vascular smooth muscle contraction” and the “cGMP-PKG signaling pathway” in the aortas of AS patients (Li et al., 2023a). In healthy arteries, vascular SMCs express contractile proteins, whose expression decreases upon vascular injury or during AS development (Grootaert & Bennett, 2021). In the EA group, multiple vascular SMC contractile markers (*e.g*., *Myh11* and *Rock2*) (Grootaert & Bennett, 2021; Li et al., 2012) showed increased expression, suggesting a possible association with vascular contractile function. The “cGMP-PKG signaling pathway” has been reported to exert vasodilatory effects (Kang, Kang & Shin, 2013). In this study, enrichment of genes related to the cGMP-PKG pathway and increased cGMP levels were observed in the EA group, suggesting a possible link to vascular function. However, the functional significance of these observations remains to be established. GO and KEGG enrichment analyses (Top 15 terms) of liver and aortic transcriptomes showed no overlapping pathways between QA and EA, suggesting that the two compounds may affect different biological processes. This raises the possibility that co-administration could produce synergistic effects, which should be tested using different dosage ratios.

Several limitations should be noted: first, only a single i.p. dose (5 mg/kg) of QA and EA was tested, without dose-response data or a positive control (*e.g*., statins), precluding direct comparison with established therapy. Second, although transcriptomic findings were supported by qPCR and cGMP measurements, mechanisms were assessed only at the mRNA level, and functional studies are needed to establish causality. Nevertheless, this study provides the first preclinical evidence that QA and EA attenuate atherosclerosis. Thus, the conclusions remain preliminary and require further validation.

## Conclusions

In summary, our study demonstrates that both QA and EA effectively inhibit ox-LDL-induced lipid accumulation and LPS-triggered inflammation *in vitro*. In animal models, both compounds alleviated the progression of AS, with improvements in lipid metabolism and inflammatory markers. At the tested dose, EA exhibited slightly superior efficacy in mitigating inflammation. Transcriptomic profiling revealed distinct gene expression patterns between QA and EA. Specifically, genes related to “Cell adhesion molecules” and immune-related pathways were downregulated in the aorta of QA-treated mice. For EA, enrichment was observed for genes associated with “Circadian rhythms” in both the liver and aorta, as well as genes involved in “Vascular smooth muscle contraction” and the “cGMP-PKG signaling pathway” in aortic tissues. Collectively, these findings suggest that QA and EA may represent promising candidates for the prevention of CVDs.

## Supplemental Information

10.7717/peerj.21907/supp-1 Supplemental Information 1 MIQE checklist.

10.7717/peerj.21907/supp-2 Supplemental Information 2 Primer sequences used for real-time quantitative PCR.

10.7717/peerj.21907/supp-3 Supplemental Information 3 Transcriptome data (liver).

10.7717/peerj.21907/supp-4 Supplemental Information 4 Transcriptome data (aorta).

10.7717/peerj.21907/supp-5 Supplemental Information 5 ARRIVE Checklist.

10.7717/peerj.21907/supp-6 Supplemental Information 6 ARRIVE Statement.

## Additional Information and Declarations

### Data Availability

The following information was supplied regarding data availability:

The raw measurements are available in the Supplemental Files
