# Supplementary material for: Quillaic acid and echinocystic acid attenuate atherosclerosis: evidence from in vitro and in vivo studies
Source: PeerJ. 2026 Nov 12;14:e21907. doi: 10.7717/peerj.21907 (PMC13770418; doi:10.7717/peerj.21907)
Supplement: Supplementary file 2 [file peerj-14-21907-s002.docx]

**Table S1** Primer sequences used for real-time quantitative PCR

| **Gene** | **Forward Primer** | **Reverse Primer** |
| --- | --- | --- |
| CD36 | ATGGGCTGTGATCGGAACTG | GTCTTCCCAATAAGCATGTCTCC |
| SR-A | TGAACGAGAGGATGCTGACTG | TGTCATTGAACGTGCGTCAAA |
| ACAT1 | CAGGAAGTAAGATGCCTGGAAC | TTCACCCCCTTGGATGACATT |
| NCEH1 | TTGAATACAGGCTAGTCCCACA | CAACGTAGGTAAACTGTTGTCCC |
| ABCA1 | AAAACCGCAGACATCCTTCAG | CATACCGAAACTCGTTCACCC |
| ABCG1 | CCGATGTCCCCTTTCAGATCA | GAGCAGCGAACAGCACAAAA |
| TNF-α | ATGTCTCAGCCTCTTCTCATTC | GCTTGTCACTCGAATTTTGAGA |
| IL-1β | CACTACAGGCTCCGAGATGAACAAC | TGTCGTTGCTTGGTTCTCCTTGTAC |
| IL-6 | TAGTCCTTCCTACCCCAATTTCC | TTGGTCCTTAGCCACTCCTTC |
| IL-10 | GCTCTTACTGACTGGCATGAG | CGCAGCTCTAGGAGCATGTG |
| MCP-1 | TTAAAAACCTGGATCGGAACCAA | GCATTAGCTTCAGATTTACGGGT |
| Dbp | CGCCCACTTGGTACAGAAGG | TATAGTCTGGGCGAGTGGGT |
| Per2 | GAAAGCTGTCACCACCATAGAA | AACTCGCACTTCCTTTTCAGG |
| Per3 | AAAAGCACCACGGATACTGGC | GGGAGGCTGTAGCTTGTCA |
| Bhlhe41 | TGTGTAAACCCAAAAGGAGCTT | TGTTCGGGCAGTAAATCTTTCAG |
| Nr1d2 | TGAACGCAGGAGGTGTGATTG | GAGGACTGGAAGCTATTCTCAGA |
| Npas2 | AAGGATAGAGCAAAGAGAGCCT | CATTTTCCGAGTGTTACCAGGG |
| β-actin | GGCTGTATTCCCCTCCATCG | CCAGTTGGTAACAATGCCATGT |
