# Supplementary material for: Quillaic acid and echinocystic acid attenuate atherosclerosis: evidence from in vitro and in vivo studies
Source: PeerJ. 2026 Nov 12;14:e21907. doi: 10.7717/peerj.21907 (PMC13770418; doi:10.7717/peerj.21907)
Supplement: Supplementary file 3 [file peerj-14-21907-s006.doc]

**Author checklist-statement**

2b: The sample size of mice was 10 mice per group to ensure that the conclusions of the experiment could be supported.

3b: For each experimental group, most animal data were included in the analysis, unless the data were significantly biased.

3c: The data points are already visible in the Figers.

4b: The confounders were not controlled, as the order of treatments and measurements, or animal/cage location was randomized.

5: Jian Ai and Yongkang Zhou were aware of the group allocation at the different stages of the experiment.

6a: All assessed outcome indicators were compared according to control and model groups.

10: For each experiment conducted, including independent replications, the data points are already visible in the Figers.

11: The research objectives, animal species, strain and sex, key methods, principal findings, and study conclusions were provided in the Manuscript.

12: ApoE knockout in mice spontaneously develops atherosclerotic features and dietary induction accelerates the disease process. From the early stage of the disease, lipid pattern, to the late stage of plaque formation, the disease process is highly similar to that of human patients, and it is one of the most commonly used models for atherosclerosis-related studies.

16c: Mice were anesthetized with 1.2% isoflurane by inhalation, and blood was collected from the ophthalmic venous plexus, followed by cervical dislocation and execution.

17b: The limitations of this study lie in: (1) Only APOE mice were selected to explore the anti-atherosclerotic effects of QA and EA, and no other animal models were investigated; (2) The high-fat/high-cholesterol diet did not change the HDL-C level, while QA significantly decreased serum HDL-C levels, it’s hard to explain, but it’s true.

18: The results of this study have the potential to be generalized to other species, but pre-tests (safety experiments) are needed to determine reagent dosages.

19: All authors declare that the study protocol was developed in advance and that the experiments were carried out in strict accordance with the developed protocol.

20: All authors declare that the study data are available and can be obtained by contacting the author (Jian Ai or Xin Tang).
